# Barking up the same tree: a comparison of ethnomedicine and canine ethnoveterinary medicine among the Aguaruna

**DOI:** 10.1186/1746-4269-5-33

**Published:** 2009-11-10

**Authors:** Kevin A Jernigan

**Affiliations:** 1 COPIAAN (Comité de Productores Indígenas Awajún de Alto Nieva), Bajo Cachiaco, Peru

## Abstract

**Background:**

This work focuses on plant-based preparations that the Aguaruna Jivaro of Peru give to hunting dogs. Many plants are considered to improve dogs' sense of smell or stimulate them to hunt better, while others treat common illnesses that prevent dogs from hunting. This work places canine ethnoveterinary medicine within the larger context of Aguaruna ethnomedicine, by testing the following hypotheses: H1 -- Plants that the Aguaruna use to treat dogs will be the same plants that they use to treat people and H2 -- Plants that are used to treat both people and dogs will be used for the same illnesses in both cases.

**Methods:**

Structured interviews with nine key informants were carried out in 2007, in Aguaruna communities in the Peruvian department of Amazonas. Informants provided freelists of plants given to dogs and explained the purpose, preparation and route of administration used. For each plant, informants also described any uses for treating people. Botanical voucher specimens were collected and additional informal observations were made, accompanying people on hunting trips.

**Results:**

Out of 35 plant species given to dogs, 29 (83%) are also given to humans for some medicinal purpose, while five are used only for dogs. However, the same plant is used to treat the same illness in both humans and dogs in only 53% of the cases. Forty-three percent of plants used to treat a particular illness for both dogs and people are administered in the same manner for both.

**Conclusion:**

Results suggest that Aguaruna canine ethnoveterinary medicine is, at least partly, an independent cognitive domain. Some of the difference in plant use between dogs and people can be explained by the fact that certain diseases mentioned only apply to dogs. Although reports of canine ethnoveterinary medicine are very sparse in the literature, Aguaruna practices show some similarities with a few trends reported for other Amazonian societies, particularly, in the prevalence of the nasal route of administration, the use of plant-based psychoactives and in the importance of ants and wasps, in some form, for training dogs.

## Background

Ethnoveterinary medicine has seen increasing recognition as a distinct area of study since the publication of two important review articles on the subject [1,2]. Recent research has addressed a broad range of topics within this field, including plant-based [3,4] and non plant-based [5] preparations, folk categories of illness [6], efficacy of treatments [7] and zoopharmacognosy [8,9]. A large majority of the work has been on ruminants, including sheep [6,10], cows [5] and camels [11]. This trend is not surprising, considering the economic importance of ruminants in many societies.

However, studies focusing on remedies for hunting dogs are largely lacking. In one notable exception, Lans *et al *[12] studied hunters' remedies in Trinidad. The authors list 36 plant species and three animals used either to improve dogs' ability to track and catch game, or to treat common injuries such as snake bite, mange and sprains. A few other authors have included data on dog medicines within larger studies. Glenn Shepard [13,14] describes the use of psychoactive and other medicinal preparations among the Matsigenka of Peru. Im Thurn's [15] classic ethnography of indigenous peoples of Guiana includes several brief examples of hunting charms. The present study aims to contribute in a small way to the further development of this important but understudied aspect of ethnoveterinary medicine.

**Figure 1:**
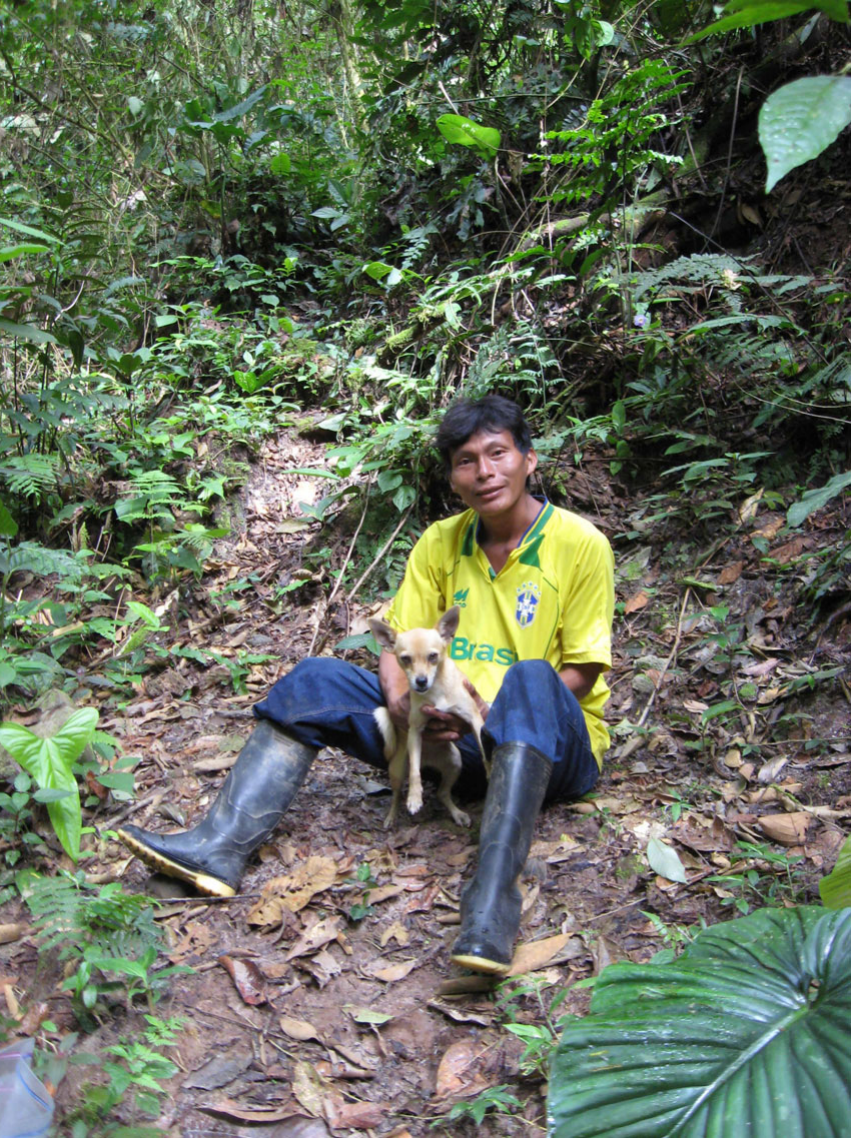
**One key informant, Agustin T. with small breed of dog used for hunting armadillos**.

The research focuses on the plants that the Aguaruna Jívaro of Peru use for treating dogs and examines whether people use the same ones in the same way to treat themselves. As in many lowland South America societies [16,17], dogs play a key role in hunting for the Aguaruna. They serve the secondary purposes of guarding homes, and to some extent, companionship. Dogs help to track and capture the following game animals: Paca (*Cuniculus paca, **kashai ***in Aguaruna), Agouti (*Dasyprocta *spp., ***kayuk***), Acouchi (*Myoprocta prattii, **yugkits***), various armadillos (*Dasypus *spp., ***shushui***) (Figure 1), White-lipped Peccary (*Tayassu pecari*, ***yugkipak***), Collared Peccary (*Pecari tajacu*, ***paki***), Southern Amazon Red Squirrel (*Sciurus spadiceus*, ***waiwash***), Gold Tegu (*Tupinambis teguixin*, ***iwan***), and various species of turtle (***kugkuim***).

Due to decreasing availability of preferred game species, people in the study communities now also hunt some animals that they did not formerly. These include Red Brocket Deer (*Mazama americana*, ***japa***), considered one of the forms that the soul of deceased person can take, and Lowland Tapir (*Tapirus terrestris*, ***pabau***), thought to be dirty because they defecate in water.

The Aguaruna traditionally hunted with ***uum ***-- 'blowguns' and still do rarely. They put ***tseas ***-- poison made from local plants in the genera *Potalia *and *Strychnos *(Loganiaceae) on the darts [18]. When a hunter would hit an animal, especially a large one, it would often run for a distance before the poison took full effect. Dogs would track down the body. Currently, most hunters use guns and dogs still play a role tracking wounded animals. When a dog is chasing an animal, the owners encourage it by shouting after it. Men yell "**esaisaik**," but women say "***estai***." To call the dog back, they shout "***biaag***, ***biaag ***and the dog's name."

Informants described how dogs learn to hunt animals. Young dogs start by chasing after small birds, and begin pursuing agoutis, as they grow. Mature dogs tend to stick with one particular animal, although they can be trained to change their focus. Accordingly, Aguaruna families try to keep five to eight dogs to hunt a variety of game. Im Thurn [15] notes that indigenous peoples of Guiana also trained each dog they owned to hunt a specific animal.

Dogs play an important role in trapping animals inside their dens. The success of this technique depends on the animal in question. Agoutis and armadillos, for example, remain in their burrows, and the hunter digs them out with a sharp stick. Pacas are more difficult to trap this way, because they emerge quickly and, being adept swimmers, head for streams or lakes. Aguaruna informants explained that paca dens have three holes, so they can always escape out one.

In the course of hunting, dogs commonly suffer from cuts and skin infections. They can be injured by some animals, particularly pacas, peccary and Ring-tailed Coati (*Nassua nassua, **kushi***) [18], as well as **dapi -- '**venomous snakes' and a variety of wasps and ants. Dogs are also prone to parasites and other digestive ailments that interfere with their hunting ability.

This work aims to address hunting medicines within the larger framework of Aguaruna ethnomedicine. McCorkle [1] points out that ethnoveterinary studies have not often compared medicines given to animals with those given to people, even though it seems natural to do so. Wanzala *et al *[2] also argue for the need to make the study of ethnomedicine and ethnoveterinary medicine complementary. Hirschkind [5: 290] notes, from her work in highland Ecuador, that " [d]omestic animals are understood to share many human characteristics, needs and desires." Gradé *et al *[19] argue that sheep owned by Karamojong pastoralists of Uganda self medicate by grazing on certain local plant species. Part of their argument rests on the fact that the Karamojong use many of the same species medicinally themselves.

The idea for the present study came while I was doing research for my dissertation [20], on the subject of how Aguaruna men identify trees. I was struck by the fact that tree species reported to have a medicinal effect for people were also often said to be useful for treating dogs. Indeed many of the kinds of illness that hunting dogs commonly suffer, such as gastrointestinal problems, cuts and snake bites are also common problems for human hunters. I began to wonder whether people always give dogs the same remedies that they take themselves or whether there are some medicines only for dogs.

Although dogs are known to have been present in some locations in coastal and highland South America before European contact [21], there is no such evidence for the upper Marañón, where the Aguaruna live. More generally, dogs are usually assumed to be a Spanish introduction in the Amazon basin [22]. If dog medicines represent a fairly recent domain of knowledge for the Aguaruna, it would seem reasonable to assume that this domain is highly derived from Aguaruna ethnomedicine in general. Accordingly, this research tests the following hypotheses: **H1 -- Plants that the Aguaruna use to treat dogs will be the same plants that they use to treat people **and **H2 -- Plants that are used to treat both people and dogs will be used for the same illnesses in both cases**.

## Methods

**Figure 2:**
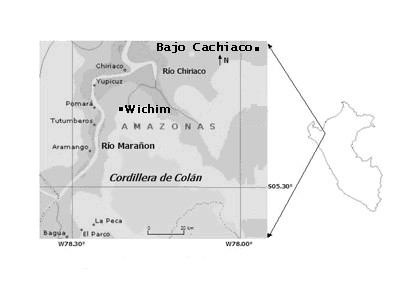
**Map of the study area**.

Some preliminary work for this project was carried out in 2004, in the Peruvian department of Amazonas, in the Aguaruna communities of Bajo Cachiaco and Wichim, on the Nieva and upper Marañón rivers, respectively (Figure 2). The main portion of the study took place in the same two communities during fall, 2007. Study communities are in the eastern foothills of the Andes, at elevations range from 230 m to 500 m above sea level, with nearby mountains up to 1000 m or so [23]. The sites include tropical wet forest and premontane tropical rainforest [24]. Both participating communities have a largely subsistence based economy involving swidden horticulture and supplemented extensively by raising of livestock, gathering of wild plants, fishing and hunting of birds and game [25].

Nine Aguaruna informants (eight men and one woman) took part in interviews and two Aguaruna field assistants worked with me to further clarify and add to those data. I obtained verbal prior informed consent (PIC) from each participant, and the research followed ethical guidelines adopted by the American Anthropological Association [26].

For each interview, I obtained a freelist of relevant plants by asking '***Wait aneasam adaikata ashi ikamnum ayau yawaa jakui tsuamatai'***. -- 'Please tell me all the plants you know that are good for treating sickness in dogs.' Informants mentioned 34 plants total. One person also described several (unidentified) species of wasp larvae. The average freelist had nine plants. For each plant named, I asked informants to describe the canine illnesses or conditions it is used to treat and the preparation method, including part used and route of ingestion. I also asked the same questions regarding any uses for people. I gained additional information about dog medicines and hunting practices in informal conversations with community members and by accompanying them during hunting trips in the forest. Botanical voucher specimens were collected for 29 plants. These were deposited in the herbarium of the Universidad Nacional Mayor San Marcos. Ethnobotanical data from Berlin [27] and collaborators helped to clarify the botanical identity of four additional species, while another remains uncollected. Finally, one plant name corresponds to mosses in general, so no collection was made.

## Results and Discussion

Informants mentioned 34 plants, corresponding to nineteen botanical families, given to dogs to improve their hunting ability or to treat illness. I will discuss plants of the former category in the first subsection. The second subsection deals with five types of digestive problems, while the last discusses treatment for ten miscellaneous illnesses.

### Medicines for Improving Hunting Ability

**Figure 3:**
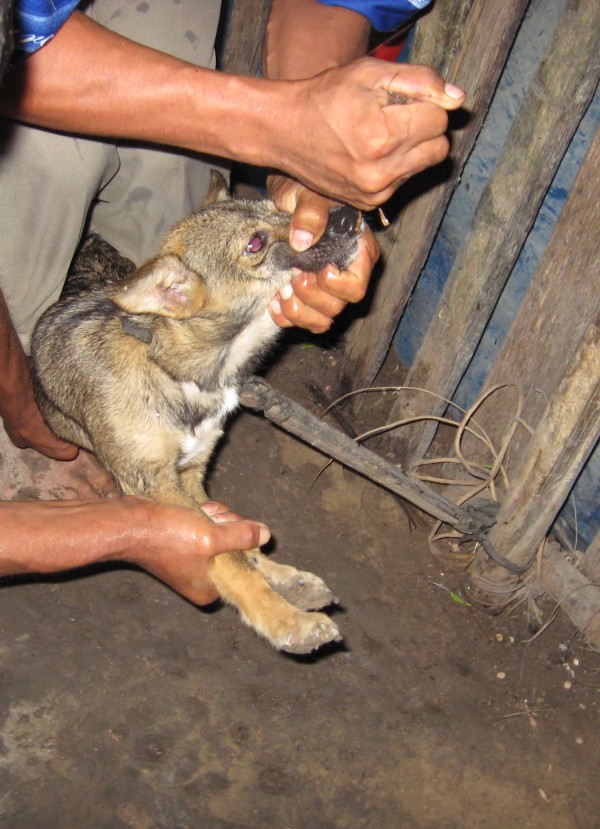
***Ampagpag *(*Piper *sp.) being administered nasally to improve hunting ability**.

The most common reason for giving dogs plant-based preparations was ***kuntinu shiig maatitusan --***'in order to hunt animals well' (Table 1). Some plants in this category are placed in the dog's nose and mouth to clean out solid white mucous deposits and heavy saliva that hinder their ability to smell game animals (Figure 3). Many of these are strong smelling species in the Bignoniaceae, Lauraceae, Piperaceae and Zingiberaceae. Aguaruna hunting dogs tend to suffer from ant and wasp bites that cause their eyes to cloud over, making their sense of smell all the more essential for finding animals. Informants also stated that dogs can lose their sense of smell, if someone lets chickens eat a piece of meat from any animal hunted by dogs.

**Table 1:** Plants used to improve hunting ability

**Family**	**Species**	**Vouch**.	**Aguaruna Name**	**Parts Used**	**Administration Method**	**SUP^a^**
Acanthaceae	not determined	J305	***tsumbaik***	roots, leaves	fed	y-
Apocynaceae	*Tabernaemontana sananho *Ruiz & Pav	J291	***kunakip***	bark, roots	inhaled in mouth, nose, fed	y-
Araceae	*Caladium *sp.	J297	***ushu***	leaves	fed	n
Araceae	*Dracontium *sp.	J293	***uchi santanik***	roots	fed	n
Bignoniaceae	*Mansoa *sp.	J298	***kaep***	leaves, roots, bark, stem tips	inhaled in mouth, nose, fed	y+
Cyperaceae	*Cyperus *sp.	J296	***yawaa pijipij***	roots	fed	n
Lamiaceae	not determined	J299	***chiujip***	leaves	fed	y+
Lauraceae	*Nectandra cuneatocordata *Mez	J171	***mantaga***	roots	inhaled in mouth, nose	n
Lecythidaceae	*Couroupita subsessilis *Pilg.	J68	***shishim***	bark	inhaled in mouth, nose	y+
Piperaceae	*Piper *sp.	J294	***ampagpag***	roots, leaves	inhaled in mouth, nose, fed	y+
Piperaceae	*Piper *sp.	J320	***shishig***	leaves	fed	n
Zingiberaceae	not determined	J317	***chiag***	leaves	inhaled in mouth	n
-	uncollected	-	***chijum***	leaves	fed	y-
-	larvae of various wasps	-			fed	n

^a ^SUP = same use for people, 'n' indicates a plant use unique to dogs, 'y-' indicates a use common to dogs and people, but with a differing route of administration for each, 'y+' indicates a plant used for the same illness and administered in the same way for both dogs and people same way for both dogs and people

Other plants in this category are stimulating and are fed to dogs, or mixed in with their normal food. The plants ***kaep ***(*Mansoa *sp.) and ***kunakip ***(*Tabernaemontana sananho*) are a noteworthy combination, because they are said to be ***waimatai ***-- 'vision producing.' Human hunters also ingest this same mix. The Matsigenka of the southern Peruvian Amazon also give dogs preparations with *Mansoa *species, but do so for different reasons. They say that the strong odor of the plant is similar to the musky smell of peccaries, a favoured game animal [14].

One Aguaruna informant said that the strongly psychoactive ***baikua ***(*Brugmansia *sp.) can be given to dogs in an enema so that they will have visions of the animals they will kill. Other psychoactives that play an important role in Aguaruna culture include ***datem ***(*Banisteriopsis *spp.) and ***tsaag ***(*Nicotiana *spp.) [28], but those were not mentioned among plants given to dogs.

A few plants in this category are not given to people for any illness or condition. Those are ***uchi santanik ***(*Dracontium *sp.), ***ushu ***(*Caladium *sp.), ***chiujip ***(an undetermined Lamiaceae) and ***yawaa pijipij ***(*Cyperus *sp.). Im Thurn [15] describes a similar use of *Caladium *species as beenas or hunting charms fed to dogs among Carib speakers of Guiana. Aguaruna informants recognize ten or so different folk species of ***pijipij ***(*Cyperus *sp.). Most serve to treat specific illnesses in people. Only ***yawaa ***'dog' ***pijipij ***is specifically for dogs. One person also mentioned ***chigki ***'bird' ***chiujip***, a folk species of that plant given only to people to help them hunt birds. To prevent dogs from being afraid of the animals they hunt, they are tied up and fed larvae of several unidentified wasp species.

### Gastrointestinal Illnesses

Gastrointestinal illnesses are a common problem for both people and animals (Table 2). Informants distinguish between ***ijag ***-- 'diarrhea' and ***shiip ***-- 'mucousy or bloody diarrhea.' The later is also associated with parasitic amoebas [29]. The fact that different plants are used to treat ***ijag ***and ***shiip ***further demonstrates that the two are distinct categories. ***Nampich ***-- 'roundworms' are also a common ailment for both dogs and people. The most common treatment is the crushed root of ***ampagpag ***(*Piper *sp.). Like ***pijipij***, more than one variety of ***ampagpag ***is recognized and the one used to treat dogs is called ***yawaa ***'dog' ***ampagpag***. It is identified by cutting a piece of root and tasting for a distinct spiciness.

**Table 2:** Plants used for digestive illnesses

**Illness**	**Family**	**Species**	**Vouch**.^a^	**Aguaruna Name**	**Parts Used**	**Administration Method**	**SUP^b^**
**diarrhea**	Euphorbiaceae	*Croton lechleri *Müll. Arg.	J302	***ujushnum***	bark	enema	n
	Solanaceae	*Capsicum *sp.	J333	***jima***	fruits	fed	y+
	Zingiberaceae	*Zingiber officinale *Roscoe	J304	***imutai ajeg***	bark	inhaled in mouth	y-
**mucousy diarrhea**	Apocynaceae	*Himatanthus sucuuba *(Spruce ex Müll. Arg.) Woodson	J201	***shipitna***	bark	enema	y+
	Euphorbiaceae	*Hura crepitans *L.	J415	***bakaij***	sap	fed	y+
	Meliaceae	*Guarea macrophylla *ssp. *pendulispica *(C. DC.) T.D. Pennington	J74	***bichauj***	bark	enema	y+
**lack of appetite**	Apocynaceae	*Tabernaemontana sananho *Ruiz & Pav.	J291	***kunakip***	bark	fed	n
	Bignoniaceae	*Mansoa *sp.	J298	***kaep***	bark	inhaled in mouth	n
	Cyperaceae	*Scleria *sp.	J301	***kujigkin***	leaves	fed	n
	Euphorbiaceae	*Croton lechleri *Müll. Arg.	J302	***ujushnum***	sap, bark	fed	n
	Euphorbiaceae	*Hura crepitans *L.	J415	***bakaij***	sap	fed	y+
	Meliaceae	*Cedrela odorata *L.	J67	***seetuj***	bark	inhaled in mouth	n
	Moraceae	*Ficus *sp.	J328	***uji***	bark	fed	n
	Zingiberaceae	*Zingiber officinale*	J304	***imutai ajeg***	roots	inhaled in mouth	y-
**stomach ache**	Euphorbiaceae	*Hura crepitans *L.	J415	***bakaij***	sap	fed	n
	Solanaceae	*Capsicum *sp.	J333	***jima***	fruits	fed	n
**worms**	Apocynaceae	*Himatanthus sucuuba *(Spruce ex Müll. Arg.) Woodson	J201	***shipitna***	bark	enema	y+
	Euphorbiaceae	*Hura crepitans *L.	J415	***bakaij***	sap	fed	y+
	Moraceae	*Ficus *sp.	J328	***uji***	bark, sap	fed	y-
	Moraceae	*Ficus maxima *Mill.	K253	***wampu***	sap	fed	y+
	Piperaceae	*Piper *sp.	J294	***ampagpag***	roots	inhaled in mouth, nose, fed	y-

^a ^Collection numbers preceded by J indicate my own collections. K indicates a collection made by Rubio Kayap, deposited at the Missouri Botanical Garden, in St. Luis Missouri.

^b ^SUP = same use for people, 'n' indicates a plant use unique to dogs, 'y-' indicates a use common to dogs and people, but with a differing route of administration for each, 'y+' indicates a plant used for the same illness and administered in the same way for both dogs and people

People also mentioned the more generic categories ***wake najamamu ***-- 'stomach ache' and ***yujumak shiig yuachu ***-- 'not eating well' as common canine problems. Most remedies in this category are intentionally fed to dogs, but people said dogs will seek out a sedge ***kujigkin ***(*Scleria *sp.) on their own, when they feel sick to their stomach. This observation relates to another important body of ethnoveterinary medicine, that of zoopharmacology, the study of apparent cases of self medication among animals [8,9].

Not surprisingly, the oral route is most common for digestive illnesses. However, some medicines in this group are given as enemas. For many bioactive compounds, enemas are an as effective a method of absorption as the oral route, with the added advantage of avoiding nausea [30].

### Miscellaneous Illnesses

Table 3 lists a variety of other illnesses mentioned that do not fit into the above two categories. One of these, called ***yawaa jati ***-- 'dog sickness,' or ***yawaa sugkuji ***-- 'dog flu' was described as a communicable and potentially fatal disease that is unique to dogs. The symptoms include runny nose, fever and lack of appetite. The most commonly mentioned remedies for ***yawaa jati ***are ***bakaij ***(*Hura crepitans*) and ***shishim ***(*Couroupita subsessilis*). Dogs also suffer snake bites or scorpion stings while hunting. Both are treated with the clear caustic sap of ***bakaij***. One must be careful not to get the liquid in the eyes, since it can damage them. Bites from ants and wasps in the eyes can be treated with juice from the stems of ***tiig ***(*Cyclanthus bipartitus*). The sap of ***ujushnum ***(*Croton lechleri*) is used to treat anemia.

**Table 3:** Plants used for miscellaneous illnesses

**Illness**	**Family**	**Species**	**Vouch**.^a^	**Aguaruna Name**	**Parts Used**	**Administration Method**	**SUP^b^**
**dog flu**	Euphorbiaceae	*Croton lechleri *Müll. Arg.	J302	***ujushnum***	sap	fed	n
	Euphorbiaceae	*Hura crepitans *L.	J415	***bakaij***	sap	fed	n
	Lecythidaceae	*Couroupita subsessilis *Pilg.	J68	***shishim***	bark	inhaled in mouth, fed	n
	Meliaceae	*Cedrela odorata *L.	J67	***seetuj***	bark	inhaled in mouth	n
**snake bite**	Euphorbiaceae	*Hura crepitans *L.	J415	***bakaij***	sap	topical	y+
**scorpion sting**	Euphorbiaceae	*Hura crepitans *L.	J415	***bakaij***	sap	topical	n
**ant or wasp sting in eye**	Cyclanthaceae	*Cyclanthus bipartitus *Poit. ex A. Rich	B1949	***tiig***	stem	topical	n
**anemia**	Euphorbiaceae	*Croton lechleri *Müll. Arg.	J302	***ujushnum***	sap	fed	y+
**dermatitis**	Apocynaceae	*Tabernaemontana sananho *Ruiz & Pav.	J291	***kunakip***	sap	topical	y+
	Commelinaceae	not determined	J303	***bicha***	leaves	topical	y+
	Rutaceae	*Citrus *sp.	J407	***yumug***	fruit juice	bath	y+
	Solanaceae	*Solanum *sp.	J418	***kukuna***	fruit juice	bath	y+
**lice**	Fabaceae	*Lonchocarpus utilis *A.C. Sm.	J419	***timu***	root	bath	n
**cuts**	Araceae	*Philodendron *sp.	B1848	***chuju daek***	stem tips	topical	y+
	Arecaceae	not determined	Bo94	***kampanak***	leaves	topical	y+
	Euphorbiaceae	*Croton lechleri *Müll. Arg.	J302	***ujushnum***	sap	topical	y+
	-	various mosses	-	***juu***	leaves	topical	y+
**swelling**	Fabaceae	*Ormosia *cf. *coccinea *(Aubl.) Jacks.	J71	***tajep***	bark	topical	y+
	Piperaceae	*Piper *sp.	J23	***untuntup***	leaves	topical	y+
**witchcraft**	Cyperaceae	*Scleria *sp.	J301	***kujigkin***	leaves	fed	n
	Zingiberaceae	*Zingiber officinale*	J310	***tunchi ajeg***	root	fed, enema	y-

^a ^Collection numbers preceded by J indicate my own collections. Bo indicates a collection made by J.S. Boster, B indicates Brent Berlin. deposited at the Missouri Botanical Garden, in St. Luis Missouri.

^b ^SUP = same use for people, 'n' indicates a plant use unique to dogs, 'y-' indicates a use common to dogs and people, but with a differing route of administration for each, 'y+' indicates a plant used for the same illness and administered in the same way for both dogs and people

Wounds, sprains and skin infections are common problems for hunting dogs. The illness ***tejemach ***corresponds to dermatitis and stems from fungal infections in some cases [29]. It is treated with bathes or topical application of medicinal plants. Dogs with lice are treated with the root of the common fish poison ***timu ***(*Lonchocarpus utilis*), but that is considered too strong for people.

Dogs can be the victims of witchcraft, just like humans. Despite the fact that ***ajeg ***-- 'ginger' (*Zingiber officinale*) is not native to Amazonia, the Aguaruna recognize ten or so folk species of the plant. Shepard (pers. comm.) has suggested that the Aguaruna may consider *Zingiber *to be an analogue of the native genus *Cyperus*, also with aromatic tubers. One folk species of ginger, ***tunchi ***'witch' ***ajeg***, serves specifically to treat witchcraft in both people and dogs. Other folk species are said to have different medicinal purposes. Informants noted that the folk species are morphologically indistinguishable, nor was I able to find any physiological distinction. Women who plant them simply have to remember where in their field they put each one.

Dogs are normally fed manioc and leftover bones and scraps. However, for the Aguaruna, taking medicine often includes some kind of restrictive diet, where certain foods and activities are forbidden for a period of time varying with the seriousness of the illness. When ill, dogs are made to avoid hot foods, roasted manioc and fish killed with ***timu ***(*Lonchocarpus utilis*) fish poison. A boy or unmarried young man must give the dog the remedy.

Overall, the results give fairly strong support to the first research hypothesis, that plants used to treat dogs will be the same ones used to treat people. Out of the 35 species given to dogs, 29 (83%) are also given to humans for some purpose. The remaining six, ***yawaa pijipij ***(*Cyperus *sp.), ***kujigkin ***(*Scleria *sp.), ***uchi santanik ***(*Dracontium *sp.), ***ushu ***(*Caladium *sp.), ***chiujip ***(an unidentified Lamiaceae) and ***timu ***(*Lonchocarpus utilis*) are unique to canine medicine. The second study hypothesis, that plants used to treat both people and dogs will be used for the same illnesses for both is only partly supported. Informants described a total of 58 uses for the 35 plants. For 31 of those (53%), the same plant is used to treat the same illness in both people and dogs. In 25 out of 58 cases (43%), plants used to treat a particular illness for both dogs and people are administered by the same route for both. These results suggest that Aguaruna ethnoveterinary medicine is, at least partly, a separate cognitive domain from ethnomedicine.

Examining the data in Tables 1, 2 and 3, three trends appear to explain most of the differences in how plants are used for treating dogs and people. First of all, some illness categories apply only to dogs. People do not contract ***yawaa sugkuji ***-- 'dog flu,' nor (with the unfortunate exception of the author) get ant stings on their eyeballs. The category ***yujumak shiig yuachu ***-- 'not eating well' also appears to be much more salient for dogs. Taking out those three illnesses, the percent agreement between canine and human uses increases to 66%. The second trend is that dogs sometimes take medicines that are considered too strong for people. Dogs (but not people) with lice are given the fish poison ***timu ***(*Lonchocarpus utilis*). The caustic sap of ***bakaij ***(*Hura crepitans*) is also used for more conditions for dogs. Thirdly, there is simply a greater variety of medicines for dogs to improve their hunting than for people. In this area, canine medicine comes closest to being a separate cognitive domain. This trend differs from some other Amazonian societies. The Matsigenka, for example, have a larger number of plants for improving the hunting ability of people than for that of dogs (Shepard, G pers. comm.).

Aguaruna canine ethnoveterinary medicine shows some important similarities with that reported for other Amazonian societies. First of all, the use of ants and wasps in some form to improve dogs' hunting ability appears to be widespread. The specific method of preparation differs cross-culturally. While the Aguaruna feed wasp larvae to dogs, Shepard [13] writes that the Matsigenka mix ants and wasps with other ingredients in preparations taken nasally. Hunters in Trinidad [12] put a wasp species with other ingredients in rum and use that in stimulating bath. Im Thurn [15] notes that hunters in Guiana sometimes made their dogs inhale smoke of a burning mixture of substances, including ants and wasps. Presumably, such practices are symbolic, intended to give dogs the same fierceness that the ant and wasp species are thought to possess. Posey [31] mentions that Kayapó boys in Brazil boys were stung by painful *Paraponera clavata *ants in a coming of age ceremony and that warriors would seek to be stung by the aggressive social wasps *Polybia liliacae*. Balée [32] has also noted a cross-cultural commonality of ant stings in initiations among speakers of languages in the Tupi Guaraní family.

The nasal ingestion route is another cross-cultural commonality. As in Trinidad [12], the purpose for the Aguaruna is to clean out dogs' noses to help them smell animals better. Shepard [13], on the other hand, speculates that strong-smelling plants administered in this way may be similar to the odor of game animals.

Both the Matsigenka [13] and the Aguaruna use psychoactive preparations to make dogs better hunters. The former give dogs *Brugmansia*, *Brunfelsia *and *Juanulloa *(Solanaceae). This fits with the emphasis that both cultures place on psychoactive plants as a means of heightening the senses.

Lans *et al *[12] report some broad categories of treatment from Trinidad that I did not find among the Aguaruna. I saw no parallel, for example, for the stimulating plant bathes that people give dogs, nor for the remedies designed to keep dogs from chasing after forest spirits instead of real animals.

## Conclusion

The domain of canine ethnoveterinary medicine appears to be significantly influenced by Aguaruna ethnomedicine in general. However, some plants and treatments are unique to dogs, especially in the area of improving hunting ability. Striking cross cultural similarities in canine medicine between the Aguaruna and other Amazonian cultures raise the question of the relative contribution of independent discovery vs. diffusion in the creation of this knowledge, which appears to postdate European contact [21,22].

Future research into Aguaruna canine ethnoveterinary medicine could explore in more detail how dogs' bodies are thought to function, specifically asking whether they have the same internal organs and physiological processes as people. With this background, one could ask more detailed questions about the pathophysiology of diseases. The fact that the Aguaruna give dogs potentially mind-altering plants to make them better hunters also raises questions about how the Aguaruna view the subjective sensory experiences of dogs [16]. Additionally, more work could be done specifically on the human side of hunting [14,33], looking at magical remedies that the Aguaruna themselves take to improve their hunting ability.

## Competing interests

The author declares that they have no competing interests.
